# Continuous Intra-arterial Infusion of Verapamil for Severe Vasospasm Treatment After Subarachnoid Hemorrhage: A Case Report

**DOI:** 10.7759/cureus.82561

**Published:** 2025-04-19

**Authors:** Debora Lopes, Bárbara Teixeira, Valter Rocha, Miguel G Santos, Elisabete Monteiro

**Affiliations:** 1 Intensive Care Unit, Unidade Local de Saúde do Médio Tejo, Abrantes, PRT; 2 Neurology, Unidade Local de Saúde Entre Douro e Vouga, Santa Maria da Feira, PRT; 3 Intensive Care Unit, Unidade Local de Saude de São João, Porto, PRT; 4 Intensive Care Unit, Unidade Local de Saúde Região de Leiria, Leiria, PRT; 5 Intensive Care Medicine, Unidade Local de Saude de São João, Porto, PRT

**Keywords:** intra-arterial infusion, intra-arterial verapamil, microcatheter, subarachnoid hemorrhage, vasospasm, verapamil

## Abstract

Delayed cerebral ischemia can occur after subarachnoid hemorrhage (SAH) and is associated with the development of symptomatic vasospasm. Despite medical treatments for vasospasm, it can be refractory. Endovascular approaches, including bolus injection or continuous infusion of verapamil via in situ placement of a microcatheter, can serve as rescue therapy. A 39-year-old male patient with a history of HIV-1 infection was brought to the Emergency Department (ED) after being found unconscious at home. He presented with a Glasgow Coma Scale score of 9 (E2V1M6), left hemiparesis, and isocoric, photoreactive pupils. A brain computed tomography (CT) revealed a large right frontotemporal intra-axial hemorrhage with ventricular system extravasation, right cisural SAH, and apparent aneurysmal dilation at the bifurcation of the right middle cerebral artery (MCA) classified as Modified Fisher (mFisher) scale score of 4, Hunt and Hess scale score of 4, and World Federation of Neurosurgical Societies (WFNS) grade IV. The patient underwent surgical intervention involving clipping of the ruptured aneurysm at the right MCA bifurcation and drainage of the right frontotemporal cisural hematoma. Treatment was initiated with nimodipine (30mg 6/6h) and milrinone infusion (1 mcg/kg/min). The initial transcranial color-coded Doppler (TCCD) indicated moderate-to-severe vasospasm in the right MCA (mean velocity [Vm] of right M1: 310 cm/s; Lindegaard Index [LI]: 5.1). On the fourth day of hospitalization (day 7 post-SAH), due to persistent moderate-to-severe vasospasm, the patient underwent therapeutic angiography with the placement of a microcatheter into an internal carotid artery (ICA) and continuous infusion of verapamil. Serial TCCD showed significant improvement in vasospasm (Vm of right M1: 176 cm/s; LI: 3.9). Despite optimized perfusion using individualized optimal cerebral perfusion pressure, the patient developed refractory intracranial hypertension, requiring the placement of an external ventricular drain and subsequent decompressive craniectomy. Endovascular bolus administration of verapamil produces transient and insufficient benefits in preventing secondary injury post-SAH. Continuous verapamil infusion may serve as an earlier and more durable alternative. This case illustrates the use of this technique, which is still not widely adopted, in a patient with severe vasospasm despite treatment with nimodipine and milrinone. Additionally, the use of TCCD as a non-invasive diagnostic and monitoring tool for vasospasm progression is emphasized.

## Introduction

Subarachnoid hemorrhage (SAH) is the third most common type of stroke, with aneurysmal rupture as the most common non-traumatic cause. The hallmark clinical presentation of SAH is sudden onset of thunderclap headaches, often accompanied by loss of consciousness, which occurs in 26% to 53% of cases [1]. Other symptoms may include neck pain or stiffness, emesis, focal cranial nerve abnormalities, seizures, or focal supratentorial deficits [1]. Clinical neurological deficits (or their absence) can be categorized by using the World Federation of Neurological Surgeons (WFNS) or Hunt and Hess scales, while imaging severity is assessed with the modified Fisher scale (mFisher) [2,3]. Among its complications, delayed cerebral ischemia (DCI) is one of the most feared and can be seen in 20% to 30% of patients with SAH. Typically developing between the 4th and the 14th days after stroke, DCI is characterized by a new neurological deterioration that cannot be attributed to other causes such as hydrocephalus, rebleeding, or seizures, and significantly worsens patient outcomes [1].

It is often associated with angiographic vasospasm, a radiological finding, but documentation of vasospasm is not a sine qua non for the diagnosis of DCI. Only half of patients with angiographic vasospasm develop ischemic symptoms, and DCI may develop without angiographic vasospasm. Recent work has suggested that other factors may contribute to DCI, such as cortical spreading depolarization, impaired autoregulation with reduced regional blood flow, intra-vascular volume contraction, and microthrombosis. Since vasospasm is a major contributor to DCI, early detection and aggressive treatment of cerebral vasospasm are critical neurocritical care interventions in SAH patients [4]. Transcranial Doppler ultrasound is commonly used as a safe, noninvasive method to measure the flow velocity of cerebral arteries and monitor for cerebral vasospasm after acute SAH. However, digital subtraction angiography remains the gold standard for the diagnosis of vasospasm in large and medium-caliber cerebral arteries [5].

Nimodipine is a cornerstone of aneurysmal SAH treatment because it has been shown to improve outcomes by decreasing the occurrence and severity of ischemic deficits [6]. In patients experiencing acute neurological decline due to vasospasm, empiric treatments include hemodynamic optimization often involving manipulation of blood pressure with vasopressors, as well as inotropic agents such as milrinone (also with cerebral vasodilatory properties) and endovascular rescue therapies such as intra-arterial vasodilator infusion and cerebral angioplasty [7,8]. There is no evidence supporting these interventions and no direct comparative studies between bolus and continuous infusion of intra-arterial verapamil. However, retrospective data suggest improved outcomes in centers offering endovascular intervention for DCI compared to those that do not.

However, there is a lack of high-quality studies to establish optimal treatment guidelines [9]. Current expert recommendations suggest reserving endovascular rescue therapies for patients with clinically symptomatic vasospasm, particularly those who do not rapidly respond to medical therapy [10]. Despite these interventions, vasospasm can sometimes remain refractory.

## Case presentation

**Figure 1:**
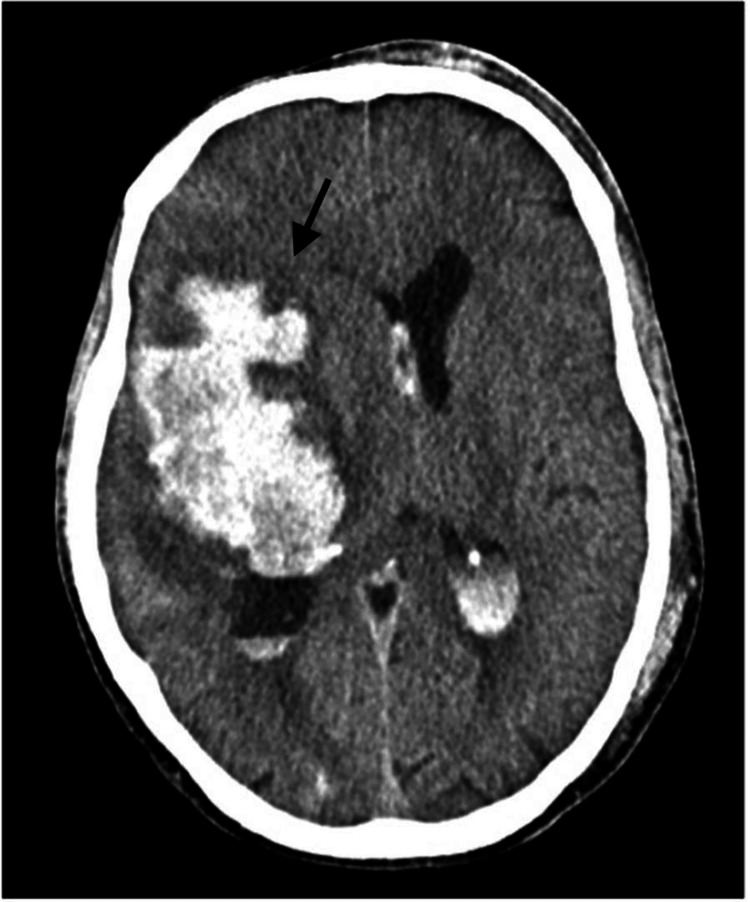
Brain computed tomography scan on admission showing right casual subarachnoid hemorrhage with intraparenchymal hemorrhagic lesion in the right temporal, insular, and frontal cortico-subcortical regions. Diffuse effacement of the cortical sulci in the right cerebral hemisphere, along with marked effacement of the right ventricle and a midline shift to the left of approximately 14 mm due to subfalcine herniation. There is also prominence of the left lateral ventricle, suggesting hydrocephalus, and intraventricular hemorrhage.

A 39-year-old man, positive for human immunodeficiency virus (HIV), was admitted at the emergency department, with deterioration of consciousness and urinary incontinence. Initial Glasgow Coma Scale (GCS) was 9 (E2V1M6) with left hemiparesis. Initial cerebral computed tomography (CT) scan (Figure 1) showed a right cisural SAH and massive right frontotemporal intra-axial hemorrhage with extravasation into the ventricular system (mFisher score of 4, Hunt and Hess score of 4, WFNS grade IV), as well as an apparent aneurysmal saccular dilatation in the right middle cerebral artery (MCA) bifurcation.

The patient underwent a microsurgical clipping of the ruptured aneurysm and right frontotemporal cisural hematoma drainage and was admitted to the neurocritical care unit (NCCU) after the procedure. Oral nimodipine (60 mg every 4 hours) and milrinone perfusion (1mcg/kg/min) were started. An EEG was also performed for two hours at admission, showing no epileptic activity.

During the first days after admission, the patient developed a progressive increase of intracranial pressure (ICP), and sequential transcranial color-coded Doppler (TCCD) ultrasonography was performed documenting right MCA severe vasospasm (Table 1). Taking into account the severe vasospasm, an attempt was made to resolve it by performing a therapeutic angiography in which 48 mg of intra-arterial verapamil was administered in bolus; however, there was only a small improvement.

**Table 1:** TCCD during the first two weeks of hospitalization. LI, Lindegaard Index; M1, cerebral middle artery; TCCD, transcranial color-coded Doppler; Vm, medium velocity

		Day 3	Day 5	Day 7	Day 8	Day 9	Day 10	Day 11	Day 15
Right M1 segment	Vm	310cm/s	181cm/s	208cm/s	176cm/s	181cm/s	206cm/s	171cm/s	141cm/s
	LI	5.1	4.7	6.3	3.9	4.7	6.4	4.8	4.1
Left M1 segment	Vm	196cm/s	154cm/s	235cm/s	166cm/s	154cm/s	230cm/s	108cm/s	94cm/s
	LI	3.1	4.4	5.9	5	4.4	6.0	2.9	2.8

**Figure 2:**
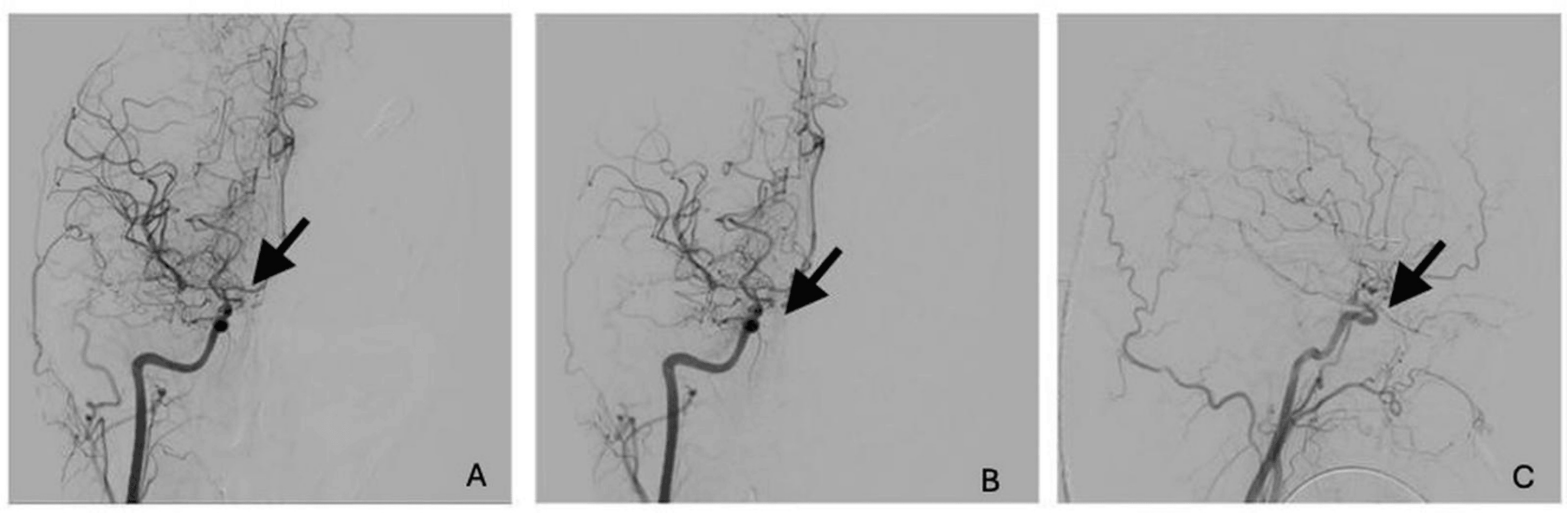
Angiography (A) before verapamil bolus and (B) after verapamil bolus infusion. (C) Angiography after microcatheter placement.

On day 4 after NCCU admission (day 7 after SAH), severe vasospasm in TCCD persisted, despite all the medical management, and a repeat angiography was performed (Figure 2). Extensive cerebral hypoperfusion was observed on the angiogram, and a continuous intra-arterial verapamil infusion was considered. A microcatheter was placed into the petrous portion of the right internal carotid artery (ICA), and an infusion of verapamil at 0.06mg/kg/h rate was started. Unfractionated heparin was also started to prevent thrombosis at an infusion rate of 500U/h, monitored according to local protocol. After the procedure, a slight improvement of vasospasm was observed (right M1 velocity 176cm/s and Lindegaard index 3.9), and no hypotension or intracranial pressure changes were assessed.

**Figure 3:**
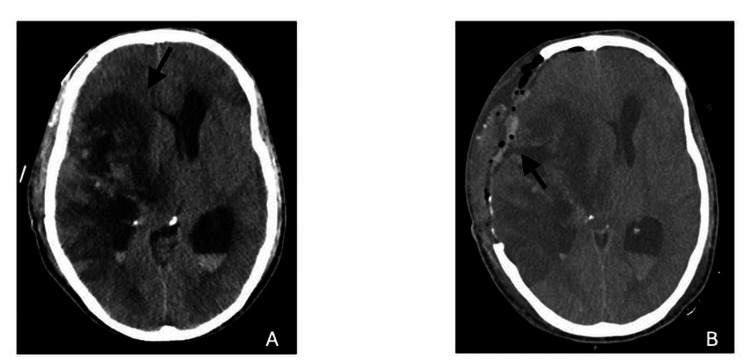
(A) Brain CT scan showing right frontal and parietal cortico-subcortical hypodensities and global increase in right frontal-temporo-parietal lesional edema. (B) Brain CT scan after decompressive craniectomy.

On day 6 (day 9 after SAH), the patient progressed unfavorably with intracranial hypertension and acute hydrocephalus, refractory to all medical measures. Verapamil infusion was stopped, and an external ventricular drainage (EVD) was placed. A brain CT scan was also performed (Figure 3), which showed right frontal and parietal cortico-subcortical hypodensities and global increase in right frontal-temporo-parietal lesional edema. Despite all the measures, intracranial hypertension was refractory and thus a decompressive hemicraniectomy was performed on day 7.

Continuous hemodynamic monitoring was maintained, and cerebral autoregulation and cerebral perfusion pressure (CPP) were optimized according to optimal CPP given continuously, at bedside, by the pressure reactivity index (PRx) and the software ICM+.

**Figure 4:**
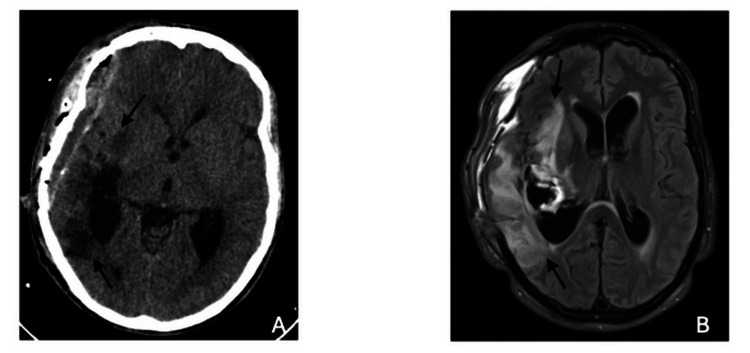
(A) Brain computed tomography scan and (B) magnetic resonance imaging showing ischemic lesions in different evolutionary stages.

On day 8, the EVD and the microcatheter were removed without complications. In the following weeks of hospitalization, the patient showed a progressive improvement of vasospasm; therefore, weaning of analgesia, sedation, and ventilation were started. Nimodipine administrations and milrinone continuous infusion were kept enterally until day 21. TCCD ultrasonography on day 22 showed flow patency in all segments studied, with acceleration in the right MCA (right M1 velocity 133cm/s and hemispheric index 3.1). Serial brain CT scans also showed improvement, with transcalvarial brain herniation reduction and significant blood reabsorption in the subarachnoid and intraventricular spaces (Figure 4).

After sedation weaning, left hemiplegia and right hemiparesis were observed with spontaneous eye-opening and carrying out orders consistently (GCS 10T, E4VTM6). A surgical tracheostomy was performed on day 39 of hospitalization, and after three days, the patient was transferred to the neurosurgery ward. After discharge, the patient began motor and functional rehabilitation treatments and was seen in a follow-up consultation approximately three months after hospital discharge. At that time, the patient had a GCS score of 15, no dysphagia, no language impairments, no cranial nerve abnormalities, and an improvement in the hemiparesis sequelae, enabling autonomous standing and walking with assistance. As of the publication of this article, the patient remains in rehabilitation and is very close to regaining the autonomy they had before this incident.

## Discussion

Prevention and management of DCI include measures to prevent cerebral vasospasm. Currently accepted treatment options to treat cerebral vasospasm are measures to optimize CPP and include early initiation of enteral nimodipine (strong recommendation), maintenance of hypovolemia (moderate recommendation), and hemodynamic augmentation if symptomatic vasospasm is present (weak recommendation) [11]. Regardless of these therapies, some patients develop persistent hypoperfusion in the context of vasospasm.

Endovascular treatment modalities are used when cerebral vasospasm is refractory to medical therapy. Two established endovascular techniques include transcutaneous balloon angioplasty (TBA) and intra-arterial administration of vasodilating substances. TBA of brain arteries is primarily used for focal proximal vessel spasm but carries a risk of arterial dissection or rupture, potentially leading to permanent vessel wall alteration with dysfunction of vasogenic autoregulation. This is particularly relevant in small caliber vessels [12,13]. Cerebral intra-arterial administration of vasodilators is one of the most commonly used endovascular modalities in our hospital. Although primarily acting locally, it carries the potential risk of systemic hypotensive effects. Other adverse effects include the risk of vessel injury, thrombosis, and hemorrhage [14]. Various agents have been used, each with distinct side effects: glyceryl trinitrate has been linked to systemic hypotension [15], papaverine to increased intracranial pressure [16], and milrinone to a high rate of vasospasm recurrence [17]. Several retrospective case series have reported positive outcomes with intra-arterial verapamil and nimodipine injection [18]. However, a major limitation of intra-arterial bolus therapy is a short and transient effect, with minimal impact on proximal vasculature [19]. The intra-arterial infusion of verapamil dilates spastic cerebral arteries [7], but achieving significant results requires high doses and several hours of infusion [19]. The advantage of verapamil over nimodipine remains unclear [20]. Reported data shows a 30% angiographic failure rate, likely due to the short action duration of such bolus [20]. Specific indications for intra-arterial verapamil infusion include peripheral and diffuse vasospasm, which may be unilateral or bilateral, although there are not enough studies to prove its benefits on the outcome of this high-risk patient group [15]. Potential complications include thromboembolic infarction, vessel dissections, air emboli, kinking or microcatheter thrombosis, induced arterial hypotension, and sepsis due to hematogenous dissemination following puncture [15].

In our patient, the initial intra-arterial verapamil bolus administration (therapeutic angiography) showed a short improvement because of the short half-life of verapamil. Verapamil continuous infusion, through a catheter placed in a petrous portion of the right internal carotid artery, was then decided, and a greater improvement of vasospasm was achieved. Despite this improvement, it was not enough to completely prevent cerebral infarctions probably due to the ongoing ischemia established prior to these interventions. No complications related to the presence of the microcatheter were reported, namely thrombus formation along the catheter, emboli, catheter dislodgement, bleeding, or infection. There have also been no reported complications associated with systemic use of verapamil, such as hypotension, bradycardia, or other cardiac conduction problems.

This report illustrates the use of continuous intra-arterial verapamil infusion as a valid therapeutic option in the treatment of refractory vasospasm when other therapies have failed, without serious adverse effects. However, we must bear in mind that continuous intra-arterial perfusion of verapamil presents some complications/limitations such as the possible development of transient hemodynamic changes, post-procedure seizures, and increased ICP with consequent decrease in CPP. These limitations contraindicate its use in patients with severe hypotension, second or third-degree atrioventricular block and sick sinus syndrome (unless a pacemaker is functioning), severe congestive heart failure (unless caused by supraventricular tachycardia), Wolff-Parkinson-White syndrome, ventricular tachycardia, and known hypersensitivity. In addition, this technique lacks established guidelines with precise indications on doses and timings for its use. To date, only case reports and one small series have discussed the utility of intra-arterial vasodilators for the treatment of vasospasm. Ospel et al. reported an additional series of 11 medically refractory cases of presumed or proven reversible cerebral vasoconstriction syndrome successfully treated with intra-arterial verapamil infusion [21].

Although intra-arterial verapamil shows promise in the management of cerebral vasospasm, especially in refractory cases, its use should be carefully considered, taking into account the possible complications and the lack of standardized protocols. The decision to use it should be individualized, based on the risk-benefit assessment for each patient, and should be taken by an experienced neuroradiologist.

## Conclusions

This case report demonstrates that continuous intra-arterial verapamil infusion could have potential benefit in the treatment of severe cerebral vasospasm when conservative treatments fail. However, further studies are required. Currently, there are no established guidelines for patient selection, verapamil dosage, timing of initiation, or duration of treatment. Developing a treatment algorithm and ensuring prompt endovascular rescue treatment in selected patients may offer a safe and effective solution for those who do not respond to conservative measures.

## References

[1] Claassen J, Park S (2022). Spontaneous subarachnoid haemorrhage. Lancet.

[2] Degen LA, Dorhout Mees SM, Algra A, Rinkel GJ (2011). Interobserver variability of grading scales for aneurysmal subarachnoid hemorrhage. Stroke.

[3] van Donkelaar CE, Bakker NA, Veeger NJ (2017). Prediction of outcome after subarachnoid hemorrhage: timing of clinical assessment. J Neurosurg.

[4] Rabinstein AA, Friedman JA, Weigand SD (2004). Predictors of cerebral infarction in aneurysmal subarachnoid hemorrhage. Stroke.

[5] Bouzat P, Payen JF, Crippa IA, Taccone FS (2016). Noninvasive vascular methods for detection of delayed cerebral ischemia after subarachnoid hemorrhage. J Clin Neurophysiol.

[6] Dorhout Mees SM, Rinkel GJ, Feigin VL, Algra A, van den Bergh WM, Vermeulen M, van Gijn J (2007). Calcium antagonists for aneurysmal subarachnoid haemorrhage. Cochrane Database Syst Rev.

[7] Sehy JV, Holloway WE, Lin SP, Cross DT 3rd, Derdeyn CP, Moran CJ (2010). Improvement in angiographic cerebral vasospasm after intra-arterial verapamil administration. AJNR Am J Neuroradiol.

[8] Mielke D, Döring K, Behme D, Psychogios MN, Rohde V, Malinova V (2022). The impact of endovascular rescue therapy on the clinical and radiological outcome after aneurysmal subarachnoid hemorrhage: a safe and effective treatment option for hemodynamically relevant vasospasm?. Front Neurol.

[9] Shah VA, Gonzalez LF, Suarez JI (2023). Therapies for delayed cerebral ischemia in aneurysmal subarachnoid hemorrhage. Neurocrit Care.

[10] Connolly ES Jr, Rabinstein AA, Carhuapoma JR (2012). Guidelines for the management of aneurysmal subarachnoid hemorrhage: a guideline for healthcare professionals from the American Heart Association/american Stroke Association. Stroke.

[11] Liu GJ, Luo J, Zhang LP (2011). Meta-analysis of the effectiveness and safety of prophylactic use of nimodipine in patients with an aneurysmal subarachnoid haemorrhage. CNS Neurol Disord Drug Targets.

[12] Jestaedt L, Pham M, Bartsch AJ, Kunze E, Roosen K, Solymosi L, Bendszus M (2008). The impact of balloon angioplasty on the evolution of vasospasm-related infarction after aneurysmal subarachnoid hemorrhage. Neurosurgery.

[13] Linskey ME, Horton JA, Rao GR, Yonas H (1991). Fatal rupture of the intracranial carotid artery during transluminal angioplasty for vasospasm induced by subarachnoid hemorrhage. Case report. J Neurosurg.

[14] Seker F, Hesser J, Brockmann MA, Neumaier-Probst E, Groden C, Schubert R, Brockmann C (2017). Pharmacokinetic modeling of intra-arterial nimodipine therapy for subarachnoid hemorrhage-related cerebral vasospasm. Clin Neuroradiol.

[15] Musahl C, Henkes H, Vajda Z, Coburger J, Hopf N (2011). Continuous local intra-arterial nimodipine administration in severe symptomatic vasospasm after subarachnoid hemorrhage. Neurosurgery.

[16] McAuliffe W, Townsend M, Eskridge JM, Newell DW, Grady MS, Winn HR (1995). Intracranial pressure changes induced during papaverine infusion for treatment of vasospasm. J Neurosurg.

[17] Fraticelli AT, Cholley BP, Losser MR, Saint Maurice JP, Payen D (2008). Milrinone for the treatment of cerebral vasospasm after aneurysmal subarachnoid hemorrhage. Stroke.

[18] Mazumdar A, Rivet DJ, Derdeyn CP, Cross DT 3rd, Moran CJ (2006). Effect of intraarterial verapamil on the diameter of vasospastic intracranial arteries in patients with cerebral vasospasm. Neurosurg Focus.

[19] Albanese E, Russo A, Quiroga M, Willis RN Jr, Mericle RA, Ulm AJ (2010). Ultrahigh-dose intraarterial infusion of verapamil through an indwelling microcatheter for medically refractory severe vasospasm: initial experience. Clinical article. J Neurosurg.

[20] Hänggi D, Turowski B, Beseoglu K, Yong M, Steiger HJ (2008). Intra-arterial nimodipine for severe cerebral vasospasm after aneurysmal subarachnoid hemorrhage: influence on clinical course and cerebral perfusion. AJNR Am J Neuroradiol.

[21] Ospel JM, Wright CH, Jung R (2020). Intra-arterial verapamil treatment in oral therapy-refractory reversible cerebral vasoconstriction syndrome. AJNR Am J Neuroradiol.

